# Deployment and Travel Medicine Knowledge, Attitudes, Practices, and Outcomes Study (KAPOS): Malaria Chemoprophylaxis Prescription Patterns in the Military Health System

**DOI:** 10.4269/ajtmh.19-0938

**Published:** 2020-04-27

**Authors:** Patrick W. Hickey, Indrani Mitra, Jamie Fraser, David Brett-Major, Mark S. Riddle, David R. Tribble

**Affiliations:** 1 Department of Pediatrics, Uniformed Services University of the Health Sciences, Bethesda, Maryland;; 2 Department of Preventive Medicine and Biostatistics, Uniformed Services University of the Health Sciences, Bethesda, Maryland;; 3 Infectious Disease Clinical Research Program, Department of Preventive Medicine and Biostatistics, Uniformed Services University of the Health Sciences, Bethesda, Maryland;; 4 The Henry M. Jackson Foundation for the Advancement of Military Medicine, Inc., Bethesda, Maryland;; 5 Department of Epidemiology, College of Public Health, University of Nebraska Medical Center, Omaha, Nebraska

## Abstract

The Deployment and Travel Medicine Knowledge, Attitudes, Practices, and Outcomes Study (KAPOS) examines the integrated relationship between provider and patient inputs and health outcomes associated with travel and deployments. This study describes malaria chemoprophylaxis prescribing patterns by medical providers within the U.S. Department of Defense’s Military Health System and its network of civilian healthcare providers during a 5-year period. Chemoprophylaxis varied by practice setting, beneficiary status, and providers’ travel medicine expertise. Whereas both civilian and military facilities prescribe an increasing proportion of atovaquone–proguanil, doxycycline remains the most prevalent antimalarial at military facility based practices. Civilian providers dispense higher rates of mefloquine than their military counterparts. Within military treatment facilities, travel medicine specialists vary their prescribing pattern based on service member versus beneficiary status of the patient, both in regards to primary prophylaxis, and use of presumptive anti-relapse therapy (PQ-PART). By contrast, nonspecialists appear to carry over practice patterns developed under force health protection (FHP) policy for service members, into the care of beneficiaries, particularly in high rates of prescribing doxycycline and PQ-PART compared with both military travel medicine specialists and civilian comparators. Force health protection policy plays an important role in standardizing and improving the quality of care for deployed service members, but this may not be the perfect solution outside of the deployment context. Solutions that broaden both utilization of decision support tools and travel medicine specialty care are necessary.

## INTRODUCTION

The U.S. military identifies malaria as among the most important infectious disease threat to the health and operational readiness of deployed forces.^1^ Annual case burden within the U.S. military varies based on the geographic and operational posture of the force, ranging from 35 to 126 in between the time period of 2009−2018.^2^ The U.S. military uses a detailed set of force health protection (FHP) policies and procedures encompassing a broad array of medical threats, to include malaria.^3–6^ Unfortunately, sporadic outbreaks of malaria remain an ongoing concern to the military leading to heightened emphasis by commanders and medical leadership makers on the use of FHP policies.^7,8^ These policies, when effectively monitored by commands, have been proven to dramatically reduce the risk of malaria during deployments.^9–11^ Malaria is also a significant medical concern to the broader beneficiary population of the Military Health System as well as those service members traveling to malaria-endemic regions while on leave.^12,13^ A previous study, conducted after a major FHP policy shift in 2009, away from the use of mefloquine as a first-line antimalarial agent, demonstrated that patterns prescribed by the Military Health System for antimalarial medications varied both as a function of the medical specialty of the ordering provider and time.^14^ Results from this study led to the question of whether this variation results from whether a provider is able to individualize their medical practice based on the needs of the patient or whether they reflexively apply policies intended for active duty military deployments to non-active duty beneficiaries (e.g., family members and retirees). A body of literature, including several reports by the National Academy of Sciences, point to the conundrum that variation in practice may reflect not only on a wide range of clinically acceptable practices with attendant potential fiscal inefficiencies but also medical errors that impact quality and patient outcomes.^15,16^ The current study analyzes data from 2012 to 2016, a time period encompassing both troop-level decline in Afghanistan, where doxycycline was the first-line prophylactic agent against malaria, and the Ebola virus disease outbreak in West Africa, leading to a large-scale deployment of troops, for whom atovaquone–proguanil was the FHP policy first-line prophylactic.^17^ This study affords the opportunity to assess antimalarial prescription pattern changes over time, as well as variability in practice patterns as a function of practice setting and practice of travel medicine as a specialty.

## METHODS

This analysis represents a line of inquiry within the Deployment and Travel Medicine Knowledge, Attitudes, Practices, and Outcomes Study (KAPOS). KAPOS examines the integrated relationship between provider and patient inputs and health outcomes associated with travel and deployments. This research was approved by the Uniformed Services University of the Health Sciences Institutional Review Board.

The Military Health System provides comprehensive medical care to 9.5 million beneficiaries, worldwide, through a network of 51 military hospitals, 424 military health clinics, and a national and international network of participating civilian TRICARE medical providers.^18^ This includes 1.4 million active duty service members, 1.7 million active duty family members, 0.8 million service members on reserve status and their families, and 5.4 million retired military and their families. The Military Health System uses an electronic medical record that sends administrative and clinical data points into an integrated administrative record system called the Military Health System Data Repository (MDR). Used to analyze health care data from both military and civilian medical facilities, pharmacies, and medical providers worldwide, the MDR also collects claims data on Military Health System beneficiaries from the civilian sites. This encompasses more than 70 million annual outpatient visits, one million hospital admissions, and 128 million prescriptions annually.^18^ The MDR and its various component data files, such as the Pharmacy Data Transaction Service, serve as the foundational data set for the clinical practice trends described in this article

All antimalarial medications prescribed from fiscal years (October through September) 2012–2016, for all patients receiving care funded by the Military Health System, regardless of location or duty status were identified. This analysis presents data on individuals aged 18 years and older. Individual medication prescriptions were identified with the following associated variables: patient age, gender, sponsor branch of service, year of prescription, beneficiary status of the patient, amount dispensed, number of refills, dispensing facility and type (e.g., civilian versus military), and the prescriber’s medical expenses and performance reporting system (MEPRS) code. The MEPRS code allows for the designation of medical specialty of the prescribing clinic from military facilities. Prescriptions from military facilities lacking an MEPRS code were excluded from provider specialty-based analyses. Both patients and medical providers were assigned unique study identification numbers for comparative analyses.

Outpatient medication prescription records for malaria chemoprophylaxis were included in the study based on the following working definitions and inclusion criteria for atovaquone–proguanil (AP), mefloquine (MQ), chloroquine (CQ), doxycycline (DX), and primaquine (PQ). Primaquine was further characterized as primary prophylaxis (PQ-1) and presumptive anti-relapse therapy (PQ-PART), which was analyzed separately. All AP and MQ prescriptions were included. Doxycycline was filtered out of the initial search if co-dispensed with either isotretinoin or topical medications typically used in the treatment of acne or rosacea, using the American Hospital Formulary Service drug class, as were the prescriptions associated with dermatology MEPRS codes. Doxycycline was further censored to include only those dispensed as one tablet daily, and those with twice-daily (therapeutic) dosing schemes were excluded. Doxycycline prescriptions of 100-mg tablets with a days supply of 36 or more days, representing the minimum number of tablets dispensed using CDC guidelines for 1 week of travel to a malaria-endemic region were included. Chloroquine prescriptions associated with rheumatology clinics were excluded because of potential indexing issues with hydroxychloroquine. Prescriptions of PQ were sorted by quantity as a proxy for indication. Primaquine in amounts less than eight tablets were excluded from the analysis. Primaquine tablet quantities of 14, 28, and 30 were designated as PQ-PART. Primaquine in other amounts were designated PQ-1. Artemether–lumefantrine and quinine prescriptions were not included in the analysis. Refills were counted as a single, index prescription.

Data analyses were performed on SAS version 9.4 software (SAS Institute Inc., Cary, NC). Descriptive statistics were generated for all variables using Pearson’s chi-square test for categorical variables. These results were then stratified by facility status and by military or beneficiary status. Facility status included military treatment facilities (MTFs) as defined by prescriptions filled at either military hospitals and clinics, or deployed medical units, and civilian facilities as defined by prescriptions from nonmilitary medical facilities, retail pharmacies, and mail order prescriptions. Study subjects were identified as “service member” if identified by codes for active duty personnel, National Guard and Reserve personnel, or “beneficiary” which would principally include military retirees and their dependents, as well as the dependents of service members. Individuals with unknown beneficiary status were removed before analysis.

Proportions of prescribed medications were categorized based on both military service status and, for MTFs, the prescribing provider’s clinic’s travel medicine expertise designation. Within the Military Health System, infectious disease (adult and pediatric), preventive medicine, and allergy–immunology clinics typically provide dedicated travel medicine services, and for analytic purposes, “Travel Medicine Specialist” clinics were considered. Pareto charts were developed to determine the proportion of chemoprophylaxis-prescribed travel medicine specialists and aggregated by either unique providers or by unique facilities. Odds ratios, with 95% CIs, were calculated to determine the magnitude of difference in malarial chemoprophylaxis medication prescriptions by facilities and travel medicine specialist by either service member or beneficiary status. Civilian facility prescriptions were used as the control comparison group between facilities and between specialist categories for comparison by medications. Odds ratio results were then plotted into a forest plot.

## RESULTS

**Figure 1.**
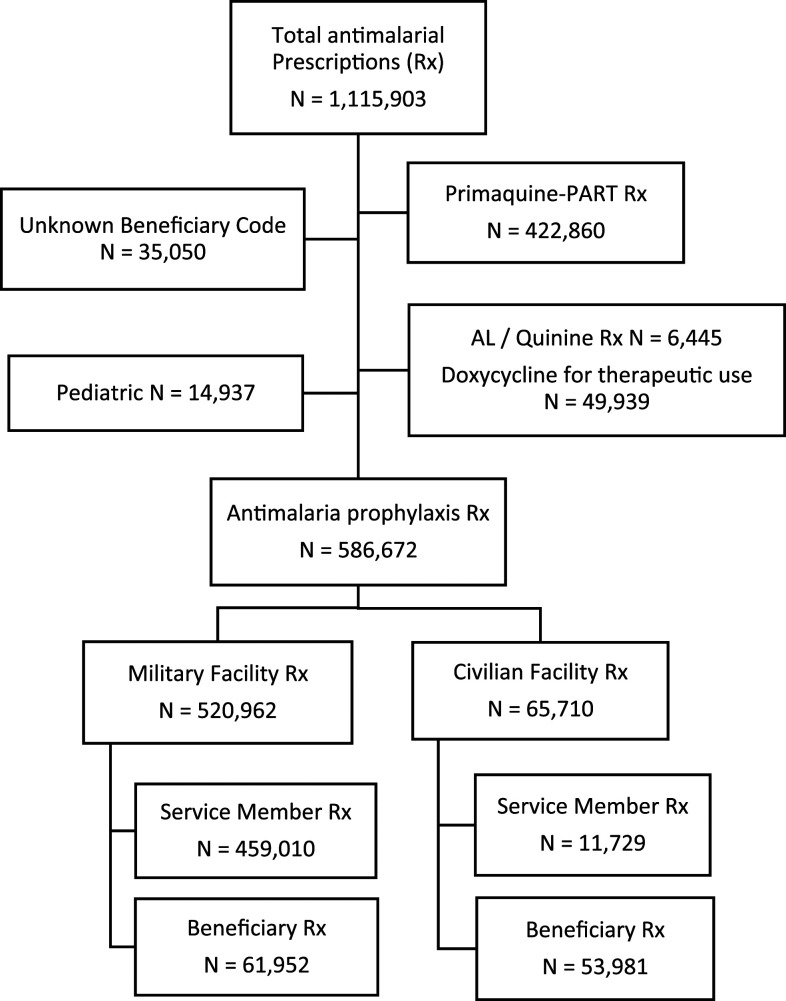
Malaria primary chemoprophylaxis prescriptions included in the analysis.

Chemoprophylaxis prescriptions were included for analysis as described in Figure 1. A total of 432,920 unique adult study subjects (Table 1) were prescribed 586,672 malaria chemoprophylaxis prescriptions for DX (74%), AP (22%), MQ (2%), CQ (1%), and PQ-1 (< 1%) during the study period from fiscal years 2012–2016 across both military and civilian medical facilities. Seventy-eight percent of study subjects (*n* = 340,657) were prescribed only one prescription for the study period. The remaining 92,263 unique individuals were prescribed more than one chemoprophylaxis with the median and interquartile range of 2 (2–3) prescriptions. The majority of prescriptions were prescribed by MTFs (89%) compared with civilian facilities (11%). Overall patient demographics consist of a majority of whom are younger than 50 years (90%) and male (82%). Military facilities have a high proportion of active duty–aged males and a relatively younger pool of beneficiaries, reflecting spouses and children of service members, compared with civilian facilities represented by smaller proportion of service members and an older population of beneficiaries. The Army is the most common military sponsor branch of service (68%), followed by Air Force (20%), and Navy/Marine Corps (11%). Other branches of the Uniformed Services that receive care in the Military Health System include Coast Guard, Commissioned Corps of the Public Health Service, and the National Oceanographic and Atmospheric Administration.

**Table 1:** Patient demographic characteristics by beneficiary category and by facility type

	Military treatment facilities (*N* = 396,911)			Civilian facilities (*N* = 36,009)		
	Service members (col %) (*N* = 359,587)	Beneficiaries (col %) (*N* = 37,324)	*P*-value	Service members (col %) (*N* = 6,555)	Beneficiaries (col %) (*N* = 29,454)	*P*-value
Age-group (years)						
18–49	352,761 (98.1)	18,676 (50.0)	< 0.0001	6,120 (93.4)	9,296 (32.0)	< 0.0001
50–64	6,615 (1.8)	11,655 (31.2)	–	433 (6.6)	8,284 (28.0)	–
> 64	211 (< 1)	6,993 (18.7)	–	2 (< 1)	11,774 (40.0)	–
Gender						
Male	316,600 (88.0)	19,255 (51.6)	< 0.0001	5,297 (80.8)	12,749 (43.3)	< 0.0001
Female	42,987 (12.0)	18,069 (48.4)	–	1,258 (19.2)	16,705 (56.7)	–
Sponsor service*						
Army	243,190 (68.0)	–	–	3,495 (53.2)	–	–
Air Force	72,363 (20.2)	–	–	1,524 (23.3)	–	–
Navy/Marine Corps	39,807 (11.0)	–	–	1,075 (16.4)	–	–
Other	2,058 (< 1)	–	–	461 (7.0)	–	–

* A total of 2,650 individuals did not have a sponsoring military service identified.

**Figure 2.**
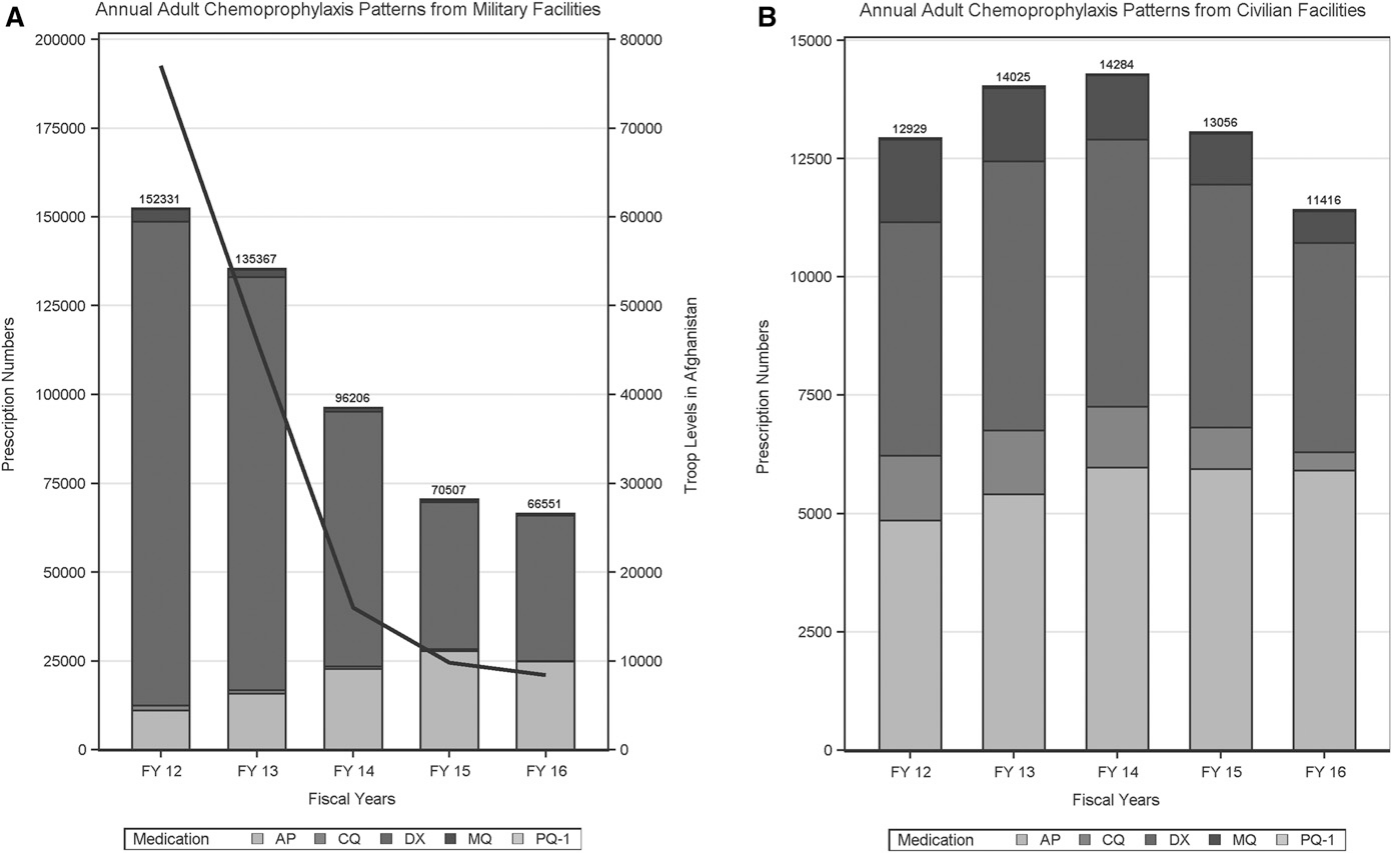
(**A**) Annual adult chemoprophylaxis patterns from military facilities. (**B**) Annual adult chemoprophylaxis patterns from civilian facilities.

Aggregate adult annual chemoprophylaxis patterns from military facilities for all five fiscal years are shown in Figure 2A. The overall decrease in total chemoprophylaxis prescriptions through the years correlates with military troop levels deployed to Afghanistan during that time period. Atovaquone–proguanil volume increases from 7.2% of total annual prescriptions in 2012–37% by 2016 and DX use shows a proportional decline from 89% to 62% during the study period. Mefloquine use declines from 2.2% of prescriptions to 0.6% by 2016. Trend changes for each antimalarial medication by fiscal year are statistically significant with *P*-value < 0.0001. Aggregate adult annual chemoprophylaxis patterns by civilian facility for all five fiscal years are shown in Figure 2B. Trend changes for each antimalarial medication by fiscal year are statistically significant with *P*-value < 0.0001 except PQ-1 (*P*-value: 0.786).

**Figure 3.**
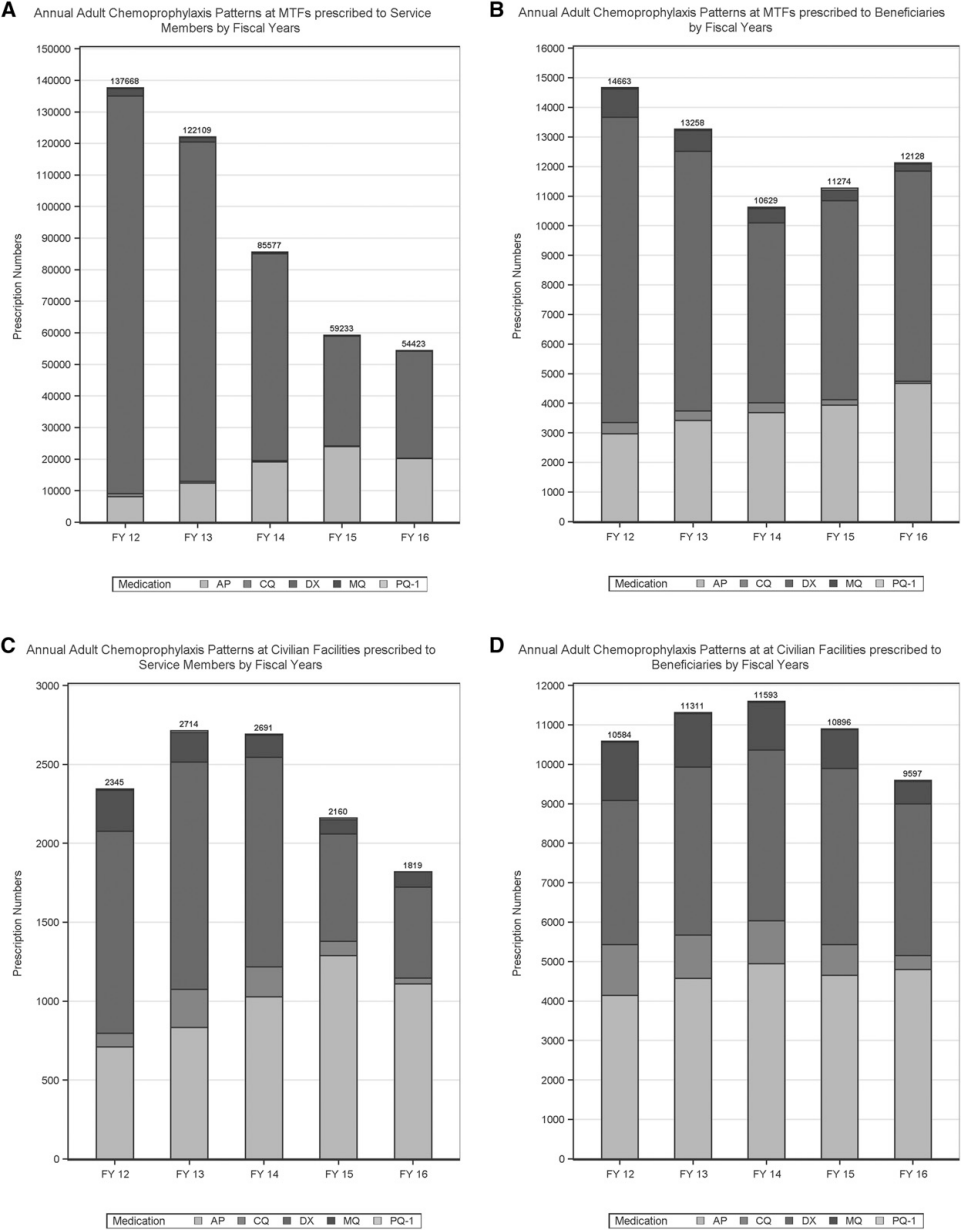
(**A**) Annual adult chemoprophylaxis patterns at military treatment facilities (MTFs) prescribed to service members by fiscal years. (**B**) Annual adult chemoprophylaxis patterns at MTFs prescribed to beneficiaries by fiscal years. (**C**) Annual adult chemoprophylaxis patterns at civilian facilities prescribed to service members by fiscal years. (**D**) Annual adult chemoprophylaxis patterns at civilian facilities prescribed to beneficiaries by fiscal years.

Comparative prescribing trends at military facilities are shown for service members (Figure 3A) versus beneficiaries (Figure 3B). Among service members, use of AP shows a steady upward trend with peak usage in fiscal year 2015. As aforementioned, DX use declines in both absolute and percent of the total but remains the majority choice for prophylaxis. Mefloquine use, already uncommon in 2012 (1%), shows a 15-fold reduction in absolute prescriptions and accounts for < 1% in 2016. Among non-service members, AP use shows a similar upward trajectory in use over time expanding from 20% to 38% of the total as DX use declines in absolute and proportional terms from 70% to 58% and MQ use at MTFs among beneficiaries also shows steady decline in usage within the study period, but remains relatively more common from 6.5% in 2012 to 2.0% in 2016.

Prescriptions from civilian sites for service members (Figure 3C) and beneficiaries (Figure 3D) show lower reliance on DX in both populations, with AP as the most commonly prescribed medication for both groups by 2016. Mefloquine use, although still in the minority, is more commonly prescribed by civilians to service members (11% in 2012–5% in 2016) and beneficiaries (14% in 2012–6% in 2016) than by military facility providers.

Military facility prescriptions were also coded by clinic specialty type allowing comparison of the proportion of prophylaxis prescription by various provider specialties. Prescriptions by MTFs without MEPRS codes numbered (*n* = 159,187 [31%]) were not included in the provider specialist analysis results. Given that 75% of these prescriptions are for doxycycline and 88% were prescribed to service members, these most likely represent prescriptions dispensed centrally by base pharmacies for deploying units. The highest volume of malarial chemoprophylaxis occurs at general primary care clinics, which are often oriented to serve the needs of active duty service members and would typically represent a mix of primary care medical specialties as well as nurse practitioners or physician assistants. In many cases, these may also be free-standing deployment mobilization clinics. A total of 198,544 prescriptions which accounts for about 62% of the total prescribed to service members at MTFs came from these clinics.

**Figure 4.**
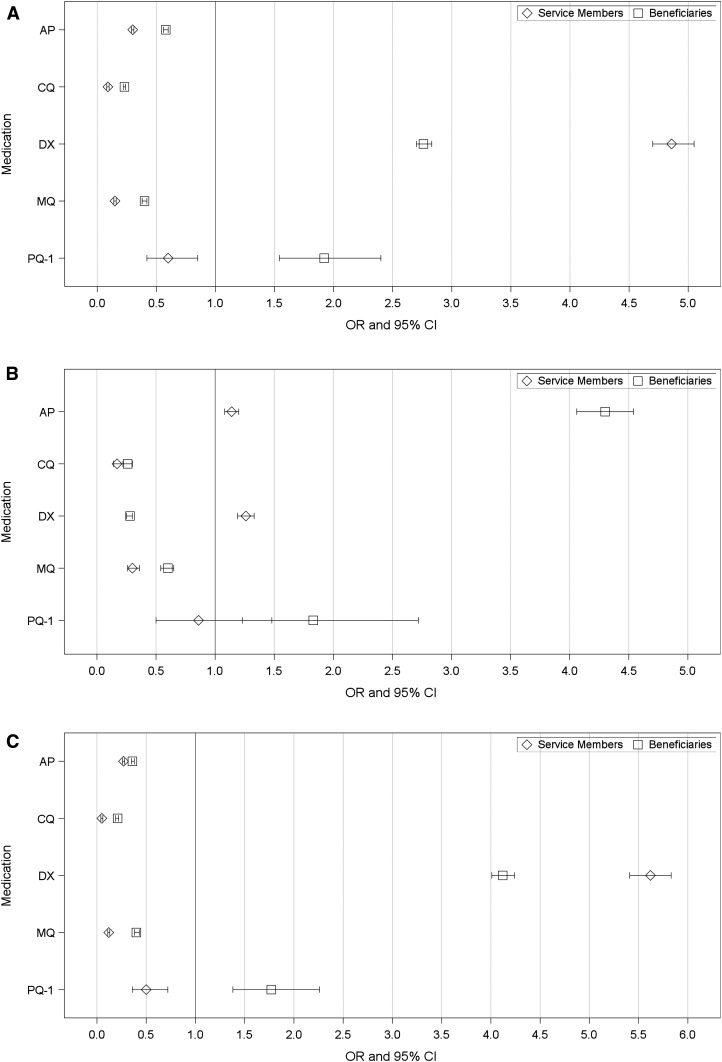
(**A**) Comparison of prescriptions by military vs. civilian facility providers. (**B**) Comparison of prescriptions by military travel medicine specialists vs. civilian providers. (**C**) Comparison of prescriptions by military travel medicine nonspecialists vs. civilian facility providers.

Odds ratios were calculated for each prophylactic medication between both service members and beneficiaries at military facilities, using civilian facilities as the control (Figure 4A, and Tables 2 and 3). The odds of prescribing DX to a service member at a military facility is higher than prescribing to a non-service member beneficiary (service members: 4.86, 95% CI: 4.70–5.05; beneficiary: 2.76, 95% CI: 2.70–2.83). Similarly, AP, CQ, and MQ all have decreased odds of being prescribed at military facilities to both service members and beneficiaries. By contrast, the total volume is low in absolute terms, PQ-1 has higher odds of prescribing at MTFs to beneficiaries (1.92, 95% CI: 1.54–2.40) but decreased odds of prescribing PQ-1 at MTFs to service members (0.60, 95% CI: 0.42–0.85). Among service members, PQ-PART was prescribed to 45.3% at MTFs compared with 9.4% at civilian facilities.

**Table 2:** Comparison of MTF vs. civilian facility prescription to service members

Medication	MTF, *N* (%)	Civilian, *N* (%)	OR (95% CI)
Atovaquone—proguanil	83,668 (18.2)	4,970 (42.0)	0.30 (0.29–0.31)
Chloroquine	2,346 (0.5)	645 (5.5)	0.09 (0.08–0.10)
Doxycycline	367,465 (80.1)	5,301 (45.4)	4.86 (4.70–5.05)
Mefloquine	4,726 (1.0)	779 (6.6)	0.15 (0.14–0.16)
Primaquine	805 (0.2)	34 (0.2)	0.60 (0.42–0.85)
Total	459,010	11,729	–
Presumptive anti-relapse therapy	380,298 (82.8)	1,224 (10.4)	7.94 (7.48–8.42)

MTF = military treatment facility.

**Table 3:** Comparison of MTF vs. civilian facility prescription to beneficiaries

Medication	MTF, *N* (%)	Civilian, *N* (%)	OR (95% CI)
Atovaquone–proguanil	18,665 (30.1)	23,109 (42.8)	0.58 (0.56–0.60)
Chloroquine	1,298 (2.1)	4,614 (8.6)	0.23 (0.22–0.24)
Doxycycline	39,002 (62.9)	20,536 (38.0)	2.76 (2.70–2.83)
Mefloquine	2,727 (4.4)	5,604 (10.4)	0.40 (0.38–0.42)
Primaquine	260 (0.4)	118 (0.2)	1.92 (1.54–2.40)
Total	61,952	53,981	–
Presumptive anti-relapse therapy	14,344 (23.2)	273 (0.1)	45.7 (40.60–51.63)

MTF = military treatment facility.

Figure 4B and Table 4 represent prescribing patterns at MTFs by travel medicine specialists compared with civilian providers as the control. Compared with civilian providers, the odds of prescribing AP by a military travel medicine specialist are higher both for beneficiaries (4.30, 95% CI: 4.06–4.54) and service members (1.14, 95% CI: 1.08–1.20). For service members, DX is prescribed at higher odds (1.26 95% CI: 1.19–1.33) than beneficiaries (0.28, 95% CI: 0.25–0.30). Travel medicine specialists at MTFs were more likely to prescribe PART to both service members (OR: 1.47) and beneficiaries (OR: 8.68) compared with the civilian providers.

**Table 4:** Comparison of MTF specialists vs. civilian facility prescriptions

Medication	Service member, OR (95% CI)	Beneficiaries, OR (95% CI)
Atovaquone–proguanil	1.14 (1.08–1.20)	4.30 (4.06–4.54)
Chloroquine	0.17 (0.14–0.22)	0.26 (0.22–0.30)
Doxycycline	1.26 (1.19–1.33)	0.28 (0.25–0.30)
Mefloquine	0.30 (0.26–0.36)	0.60 (0.54–0.65)
Primaquine	0.86 (0.50–1.48)*	1.83 (1.23–2.72)
Presumptive anti-relapse therapy	1.47 (1.36–1.60)	8.68 (7.38–10.20)

* No statistically significant results.

Figure 4C, Table 5 represent the prescribing patterns by non-travel medicine specialists at MTFs to service members and dependent/retirees, with civilian providers as the control. The odds of prescribing AP by a nonspecialist is lower for both beneficiaries (0.36, 95% CI: 0.35–0.38) and service members (0.27, 95% CI: 0.26–0.28). Military nonspecialists prescribe DX to both service members and beneficiaries at higher odds (service members: 5.62, 95% CI: 5.41–5.83; dependents/retirees: 4.12, 95% CI: 4.01–4.24) than civilian comparators but are less likely to prescribe MQ to service members (OR: 0.12) or beneficiaries (OR: 0.40). Military nonspecialists prescribe PART at significantly higher rates than civilian providers for both service members (OR: 7.42) and particularly beneficiaries (OR: 53.4).

**Table 5:** Comparison of military treatment facility nonspecialists vs. civilian facility prescriptions

Medication	Service members, OR (95% CI)	Beneficiaries, OR (95% CI)
Atovaquone–proguanil	0.27 (0.26–0.28)	0.36 (0.35–0.38)
Chloroquine	0.05 (0.04–0.06)	0.21 (0.19–0.22)
Doxycycline	5.62 (5.41–5.83)	4.12 (4.01–4.24)
Mefloquine	0.12 (0.11–0.13)	0.40 (0.38–0.43)
Primaquine	0.50 (0.36–0.72)	1.77 (1.38–2.26)
Presumptive anti-relapse therapy	7.42 (7.00–7.88)	53.4 (47.32–60.28)

A total of 14,625 unique nonspecialist providers prescribed a total of 465,357 primary prophylaxis prescriptions during the study period. The volume per provider ranges from a single provider with 6,880 prescriptions, to 4,285 providers with only a single prescription. The median number of prescriptions was 3 (IQR: 1–11). Eighty percent of nonspecialists (*n* = 11,743) wrote 10 or fewer malaria chemoprophylaxis prescriptions during the 5-year study period.

Facility-level analysis demonstrated a total of 163 unique facilities with travel medicine specialists prescribing 16,202 malarial chemoprophylaxis prescriptions (Supplementary Figure 5A). The facility with the highest number of prescriptions for the study period is from Walter Reed National Military Medical Center (WRNMMC, *N* = 2,439), which represents 15% of the total number of prescriptions by specialty clinics. Eighteen facilities represent the top 80% of the total antimalarial prescriptions among travel medicine specialists. By contrast, 339 unique facilities (Supplementary Figure 5B) were identified to have nonspecialists prescribing 345,573 malarial chemoprophylaxis prescriptions. The facility with the highest number of prescriptions are from Fort Bragg, *N* = 22,557, which represents about 7% of the total number of prescriptions by non-specialty clinics. A total of 51 facilities represent the top 80% of the total antimalarial prescriptions by non-travel medicine specialists.

## DISCUSSION

From its earliest use among allied forces in the Pacific Campaign of World War II, malaria chemoprophylaxis has been a priority for the U.S. military, leading to the dictum that ensuring adherence is not just a medical responsibility, but a command responsibility.^19^ Recent experience with U.S. military operations in sub-Saharan Africa and Afghanistan have shown ongoing problems with low adherence to doxycycline and mefloquine chemoprophylaxis, often leading to outbreaks of malaria.^7,8,20^ One notable exception to this experience is the very high rate of reported adherence and lack of cases reported among U.S. military forces while deployed to Liberia as part of the Ebola virus outbreak response Operation United Assistance in 2014–2015.^10^ Faced with a well-known, high threat from infectious diseases in this operation, the military health community provided robust, expert-level consultation on FHP measures to all deploying forces and consistently reinforced them in an ongoing manner during the deployment to a level not seen in previous deployments.^17^ This suggests that how risk management and prevention information is provided and, by whom, may have greater impact on knowledge, attitudes, and practices of deployers/travelers than merely what information is provided or what is prescribed.^21,22^

Within the Military Health System, antimalarial prescriptions are driven largely by the deployment of active duty service members and the FHP policies that determine medication selection. Reflecting the operational tempo, scale, and location of a globally deployed force, the prescription patterns during this study’s time period reflect force reductions over time in Afghanistan (where DX is the first-line agent) as well as increased operations in sub-Saharan Africa (notably for the Ebola outbreak in 2014 and 2015), where AP is the first-line agent. Particularly noteworthy is the sustained sharp reductions in MQ use by military health providers, particularly among the service members, over the past decade in response to emerging concerns about neuropsychiatric side effects and changes in the FHP policy.^14^ Aggregate prescriptions from civilian providers consistently reflect higher rates of MQ, CQ, and AP use. Although absolute numbers are small (well below 1% of the total number of prescriptions), military providers were more likely to use PQ-1 for beneficiaries than their civilian counterparts. This may reflect unique aspects of the population (including destination of travel), more detailed knowledge about travel medicine, or more generalizable prior experience with PQ as PART.

When military facility data are parsed, specialties in which travel medicine services constitute a core component of graduate medical education and routine practice, are much more likely to use AP then their generalist counterparts, particularly when caring for beneficiaries, that is, they make context-specific shifts in practice. Although all military facility medical providers have access to on-line resources (e.g., the CDC’s yellow book and Shoreland’s Travax), utilization rates are unknown, and these resources are generally neutral about ranking a preferred chemoprophylaxis agent. Rather, the dominant experience across the military as both a medical provider and as a traveler/deployer would be with deployments to Afghanistan, where DX is the standard. A shift in chemoprophylaxis choice between service member and beneficiary is not seen (Supplemental Figure 5C) among non-travel medicine specialty types, inferring that these military providers base their practice on shared experience that comes from deployment and knowledge of FHP policies into contexts for which they were not designed. This would also explain the disproportionate prescribing of PART by military providers in general, but particularly for nonspecialists to beneficiaries. A question of interest is whether broader experience with AP during operations in Africa, combined with decreasing forces in Afghanistan, will eventually lead to further increased uptake of AP across all military practice settings in the future. The impending impact of tafenoquine implementation will need assessment. Of special note, although these results suggest a more nuanced approach to clinical decision making by travel medicine specialists seeing both service member and beneficiary populations, their relative impact on the overall provision of care is comparably small, accounting for less than 1% of all chemoprophylaxis prescriptions.

Force health protection policy–driven care for service members provides a public health–oriented, evidence-based approach to risk communication with the deployer/traveler and for clinical decision-making of prevention interventions.^23^ As shown in this study, there are a number of high-volume clinical settings not staffed by travel medicine specialists. Even if one considers the delivery of care provided at high-volume mobilization sites to be “specialized,” the practitioners involved would not necessarily develop the depth and breadth of experience one might expect of someone with the intensive graduate medical education travel medicine experience of military Preventive Medicine and Infectious Disease programs, nor the Masters of Tropical Medicine & Hygiene degree and other tropical and travel medicine courses at the Uniformed Services University or similar civilian programs.^24,25^ Nor would experience at high-volume FHP policy–driven sites deploying large numbers of troops for a very specific mission profile, necessarily translate well for varied mission settings, nor mirror mastery of the depth and breadth of knowledge required of American Society of Travel Medicine & Hygiene’s Certificate of Knowledge in Clinical Tropical Medicine and Travelers’ Health (the CTropMed^®^) or the International Society of Travel Medicine Certificate program.^26,27^

Force health protection policy is designed by experts for both the itinerary, risk tolerance, and baseline health profile of active duty service members, but they are not designed for the broader population of beneficiaries. The analysis described here is not designed to assess whether the observed variation in practice represents “error” versus a wide range of acceptable practice patterns. Yet, the lack of tailoring to patient context suggests that specialists may provide higher quality care. Furthermore, one must consider whether the variation in antimalarial prescription is the proverbial “ears of the hippopotamus” representing the risk of unseen variation and disparities in other aspects of travel medicine prevention practices.^28^ Whether this equates to increased risk of harm to a patient is an important question that requires further study. Within MTFs, 50 percent of the total number of antimalarial prescriptions are written by medical providers who wrote fewer than three chemoprophylaxis prescriptions during the 5-year study period. Concern over the level of experience and applied practice in travel medicine must be taken seriously. Knowledge, attitude, and practice surveys of deployed military medical providers show as many as two-thirds made errors in prescription and management of travelers diarrhea.^29,30^ Furthermore, studies among civilian primary care practitioners consistently show gaps in travel self-efficacy, knowledge, and reported practice particularly associated with low practice volume.^31,32^ Rather than focusing on sustaining a high level of training-based proficiency, beyond knowledge of FHP, for a large (tens of thousands) number of medical providers across hundreds of medical facilities, it seems appropriate to optimize the access and use of decision support systems (e.g., Travax and the CDC’s Travelers’ Health resources). Given the facility-level data described in this report, from a health system policy perspective, it is equally or more important to ensure access to specialists in travel medicine at high-volume sites both for direct patient care and to serve as subject matter expert resources.

This study design has several limitations. First, this study did not include detailed medical record review to confirm the purpose of each prescription, reason for travel, or total duration. Because DX is not solely an antimalarial, we anticipate some misclassification bias, although it is likely non-differential. Second, it is possible we may not be capturing all prescriptions by deployed units, if not captured by the electronic medical record. Third, the determination of purpose for PQ use was abstracted based on the number dispensed, so it is possible that doxycycline for trips lasting less than 1 week was not captured, and primary prophylaxis prescriptions of primaquine for trips requiring an amount equal to PART regimens were misclassified. Finally, we were unable to determine the medical specialty of civilian facility prescribers with the data available, so unable to conduct the same degree of analyses as those within the MTFs for whom those data are available. Some handwritten prescriptions originating in a civilian facility may have been filled at a military pharmacy, or vice versa. The MDR contains multiple separate data files. The prescription data file does include a facility ID code for where the prescription was dispensed and prescribing provider demographics. Although prescribing facility information is readily available for prescriptions originating in and dispensed at military pharmacies (as discussed), these data ate not captured for “purchased care” prescriptions written outside of military facilities and filled at civilian pharmacies. Linking civilian prescriptions back to more detailed encounter data is theoretically possible but technically fraught because of the lack of a common linking variable between the two files and the lack of consistent ICD coding practice for travel medicine services. More detailed analysis of civilian provider demographics (e.g., medical specialty) would enhance the generalizability of this analysis. Unfortunately, this approach would still not allow for a practice volume–based comparison because a given civilian provider may perform at a high rate of travel medicine encounters in general but only rarely see Military Health System patients. Refining this analytic approach will be undertaken in future analyses within the KAPOS line of effort.

KAPOS has several strengths. The large sample size provided adequate power for detecting small differences in a variety of subset analyses. Inclusion of prescriptions originating from civilian facilities and of beneficiaries enhances our generalizability to the larger U.S. population. In general, longitudinal data on malaria chemoprophylaxis prescribing patterns is limited. Although longitudinal studies have recorded prescribing patterns in specialty travel clinics and participating primary care clinics through TravEpiNet, that study may not reflect broader national practice patterns, particularly in non-travel medicine specialty clinics.^33^ KAPOS provides an aggregate look at civilian prescribing patterns, which includes a variety of clinic settings such as primary care and specialty clinics. We were also able to show detailed analysis of primary care patterns separately, albeit those originating from MTFs.

It is clear that as the Department of Defense moves toward a learning healthcare system model, data provided by platform systems such as KAPOS, augmented with methods to determine appropriateness (or non-appropriateness) of deployment and travel health practices and combined with direct surveys to post-travelers/deployers, will inform and accelerate implementation of higher quality care to our service members and beneficiaries. Models of such a system have been shown to be successful with the example of the Joint Trauma System—no such system exists for non-trauma infectious disease deployment health quality practice management, but is sorely needed.^34^

## CONCLUSION

Malaria prophylaxis patterns may vary by practice setting, beneficiary status, and medical specialty. Although both civilian and military facilities prescribe an increasing proportion of AP, DX remains the most prevalent antimalarial at military facility–based practice. Civilian providers dispense higher rates of MQ than their military counterparts. Force health protection policy plays an important role in standardizing and improving the quality of care for deployed service members. Among primary care specialties, these practices appear to carry over, but this may not be the perfect solution outside of the large-scale deployment context. If in fact the practice variations described here do translate into disparate health outcomes, then solutions that broaden access to and uptake of the knowledge products or specialty care are required. Given the diffuse nature of travel medicine services represented by the high number of low-volume malaria chemoprophylaxis prescribers, facility-based solutions may be the key. Additional studies within the U.S. military context looking at the relationship between provider specialty and practice volume on self-efficacy, quality of care, and patient outcomes in this context are needed.

## Supplemental figure

Supplemental materials
