# Supplementary figures and images for: Deployment and Travel Medicine Knowledge, Attitudes, Practices, and Outcomes Study (KAPOS): Malaria Chemoprophylaxis Prescription Patterns in the Military Health System
Source: Am J Trop Med Hyg. 2020 Apr 27;103(1):334–43. doi: 10.4269/ajtmh.19-0938 (PMC7356474; doi:10.4269/ajtmh.19-0938)

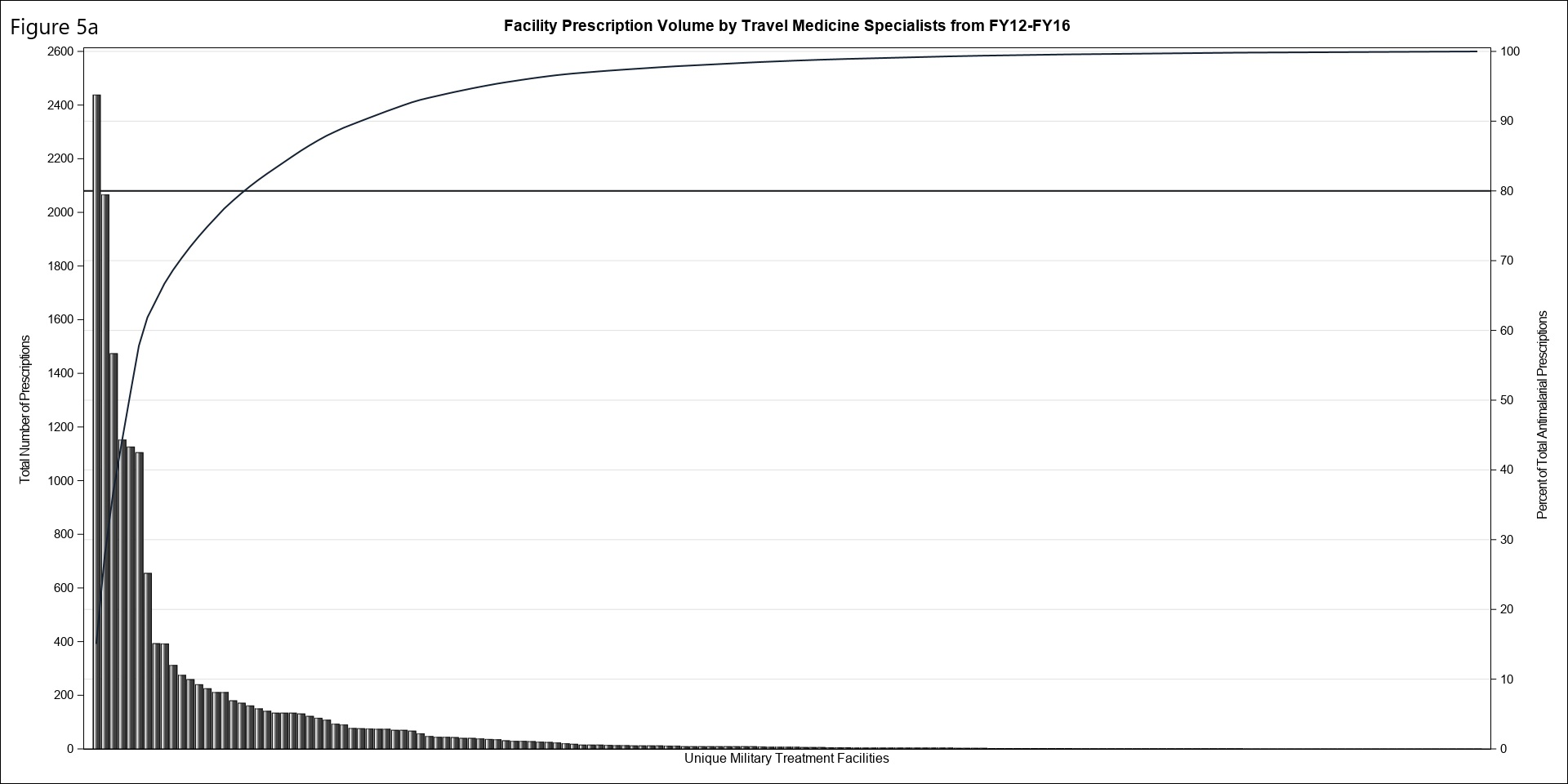

Supplement: Supplementary file 1 [file tpmd190938.SD1.tif]

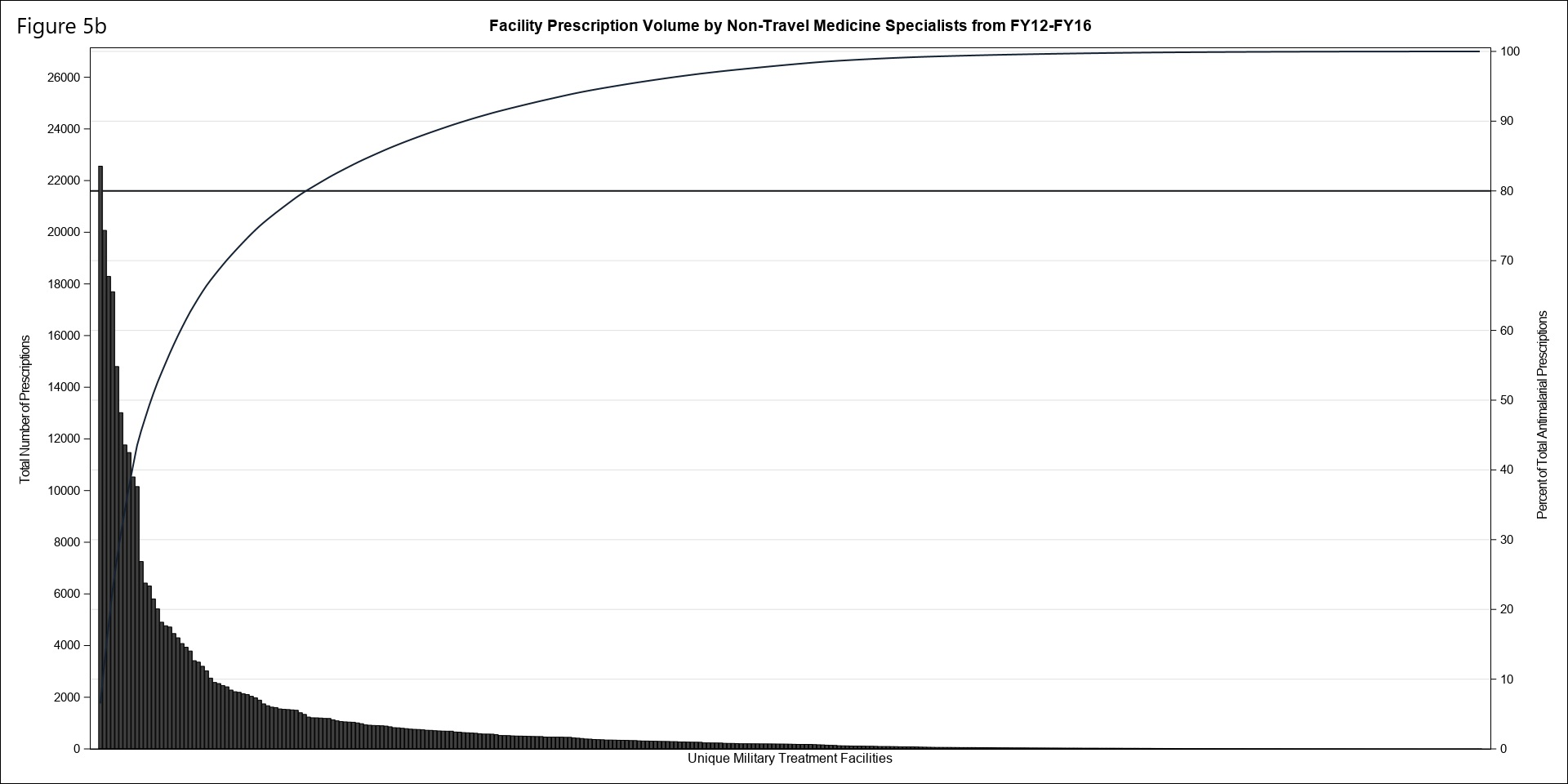

Supplement: Supplementary file 2 [file tpmd190938.SD2.tif]
